# Break-Bone Fever

**Published:** 1881-01-20

**Authors:** 


### BREAK-BONE FEVER.

A mild epidemic of this disease has prevailed in Charleston, S. C., during the past summer, and Dr. F. Peyre Porcher furnishes a report of it to the National Board of Health Bulletin. Although extensive paving had been done in the city, the fact that the disease occurred in sections of the country where such a supposable cause was out of the question leads Dr. Porcher to refer the cause to "general and wide prevailing atmospheric influences."

The symptoms vary exceedingly—some being present and some absent—as follows: The disease generally begins with a feeling of coldness, or by a chill, followed by a fever—this, with a temperature ranging from 100 to 105°, lasts generally from twenty-four to forty-eight hours, occasionally extending to four or six days, and even in rare cases to seven. Relapses occasionally, specially in those who have gone out too early. Headache frequent, generally frontal, from the beginning. Miliary eruptions, sometimes elevated and red, like measles, and the occasional presence of sudamina over the face, neck and body; sometimes the eruptions were confined to the body, and endured for days after recovery. In some cases there was slight desquamation—furfuratious or branny in character. Sweating profuse in many persons, though *often absent.* Hence some physicians are inclined to consider the disease to be *suetle miliare* of a mild form. "Break-bone" is the best name, because pain in the bones and limbs is the most constant symptom. There is often great restlessness during the fever, and in some a feeling of tightness or congestion about the throat, with bleeding in a few cases known to us. Catarrhal symptoms are rarely present, although cough has occasionally existed. Bleeding from the nose not unusual in children, and also increase in the menstrual molimen has been observed. Pain in the back and limbs markedly present, but no decided swelling of joints, no carbuncular enlargements or boils, as in the epidemic of dengue, of forty years since, or in that of "break-bone" which followed some years subsequently. Weakness and prostration have been very decided, but not nearly to such an extent as in previous epidemics. Some of the physicians consider that there has been a tendency to hepatic torpor or congestion, of no great severity, however. There have been no cases of decided jaundice. Nausea and vomiting seldom occur.

The disease does not affect all the members of the household, oftentimes only one or two being seized, though six have been taken in one house; in this respect differing from the dengue, as described by Prof. Dickson, and from the epidemic of thirty years since. Then 10,000 were down; no one was well enough or strong enough to help his neighbor, and one had to learn to walk over again.

It is difficult to calculate the number who have suffered, as very many have not employed a physician; from 2,000 to 3,000, perhaps, approximate the number.

Very little active treatment has been used, the following being that usually adopted: A mild laxative, saline or mercurial, hot teas, nitre, pediluvia, sinapisms, etc., and quinine during and after the attack, upon theoretical grounds, with occasionally mild stimulants. Several persons have recovered with no treatment whatever.

It has prevailed among both races, perhaps equally, and not a single death is ascribed to this disease as far as reported. The only disadvantage which accrues to those who take it is the time lost and the tempotary pain and weakness from which they suffer.

---

A FRENCH physician recommends the treatment of burns with oil of turpentine, covering the place with gummed goldbeater's skin.—*Boston Journal of Chemistry.*

